# Novel Aspects of Immunogenetics and Post-Transplant Events in Kidney Transplantation

**DOI:** 10.3389/ti.2024.13317

**Published:** 2024-12-05

**Authors:** Ilkka Helanterä, Salla Markkinen, Jukka Partanen, Kati Hyvärinen

**Affiliations:** ^1^ Transplantation and Liver Surgery, Helsinki University Hospital, University of Helsinki, Helsinki, Finland; ^2^ Finnish Red Cross Blood Service, Helsinki, Finland

**Keywords:** kidney transplantation, HLA matching, genome-wide association study, polygenic risk score, candidate gene analyses

## Abstract

HLA typing and matching have been crucial in kidney transplantation, but methods for assessing tissue histocompatibility have advanced significantly. While serological-level HLA typing remains common, it captures only a small fraction of true HLA variation, and molecular matching is already replacing traditional HLA matching. Recent studies have expanded our understanding of genetic tissue compatibility beyond HLA loci. Candidate gene analyses and genome-wide association studies (GWAS) have identified genetic factors linked to post-transplant complications, though replication of these findings is challenging. An alternative approach involves genome-wide matching of genes or genetic variations. This method has shown promise in hematopoietic stem cell and kidney transplantation. For instance, homozygous gene deletions in LIMS1 or complement factor H (CFH) genes have been associated with acute rejection risk. This may be due to alloimmune responses against proteins absent in the patient but present in the graft, or due to the missing protein’s function. Genetic studies in clinical medicine face challenges due to the interplay of genetic and environmental factors, necessitating large datasets for meaningful associations. International collaboration and large consortia, like iGeneTRAin, are essential for validating findings and advancing the field. This review highlights recent advancements in immunogenetics and tissue histocompatibility, emphasizing future research directions.

## Introduction

Although human leukocyte antigen (HLA) typing and matching have been key elements of kidney transplantation since the early days, the knowledge on HLA molecules and HLA matching, and methods used for assessing tissue histocompatibility have evolved enormously during the past decades. Serological-level HLA typing is still used for organ allocation in many programs, and although it is a highly simplified view of tissue histocompatibility, robust evidence still supports the association of serological HLA mismatch with kidney graft outcomes. For example, large registry reports from the Collaborative Transplant Study (CTS), including mainly European transplant centers, or from United Network for Organ Sharing (UNOS) from the US demonstrate a strong independent association between HLA-matching at the *HLA-A, -B* and -*DBR1* loci and graft and patient outcomes, independent of donor type, initial immunosuppression, or transplant era [1, 2].

With increasing frequency of high-resolution HLA typing, it is well known that traditional HLA matching captures only a small fraction of actual HLA variation. Immunogenicity of HLA molecules is mediated through epitopes, groups of amino acid residues recognized by HLA antibodies. Perhaps a biologically more plausible and relevant method to quantify differences between recipient and donor tissue types is to look at molecular mismatch of the smallest functional units of epitopes, the so called eplets [3]. Eplets are small clusters of amino acids identified on the surface across HLA molecules. Instead of directly comparing amino acid sequence differences between the whole HLA molecules, eplet or molecular mismatch can be quantified using a computer algorithm [4]. Evidence suggests that molecular mismatches, especially in HLA-DR and -DQ, are associated with many immunological outcomes after kidney transplantation [5, 6].

In addition to the traditional HLA typing or molecular matching, recent studies have shed light on more comprehensive aspects of genome level tissue histocompatibility, outside HLA genes and reaching far beyond. The aim of this review is to provide an overview of the novel aspects non-HLA-related alloimmunity, immunogenetics, and tissue histocompatibility in light of recent research findings, and discuss some future considerations that would help the field proceed forward with the ever-increasing amount of genetic information related to kidney transplant outcomes.

## Non-HLA Antibodies and Alloimmune Responses

Non-HLA-related molecules have significance in alloimmunity, as acute antibody-mediated rejection (AMR) has been described in HLA identical siblings [7], and as AMR-related changes have also been detected in patients without HLA antibodies [8]. Indeed, one way to analyze the significance of histocompatibility antigens in kidney transplantation is the ability of the recipient to produce pathological antibodies against donor tissues. Several non-HLA donor-specific antibodies (DSA) have been identified, such as angiotensin II type 1 receptor (AT1R)- antibodies [9, 10], endothelial cell antibodies [11], and others [12], but their clinical significance has been somewhat questionable [12]. Not all non-HLA antibodies are in fact allo-antibodies, e.g., AT1R antibodies are classically considered auto-antibodies.

A study by Senev *et al.* studied the clinical significance of 13 different pretransplant non-HLA antibodies and was able to show that only antibodies against Rho GDP-dissociation inhibitor 2 (ARHGDIB), a minor histocompatibility antigen, were associated with graft failure in univariable and multivariable models, with an increased risk among patients with also HLA DSA. In addition, increased intrarenal expression of the *ARHGDIB* gene was seen among patients with AMR, although none of the non-HLA antibodies were independently associated with the risk of ABMR [13]. A recent study by Carapito *et al.* investigated the role of the MICA gene matching, located near the HLA B gene in the major histocompatibility complex (MHC) segment, as a candidate histocompatibility locus. Their study showed that mismatches of MICA alleles were associated with reduced graft survival, and anti-MICA DSA were associated with ABMR [14]. Although the MICA gene is located close to HLA B gene that often is matched in kidney transplantation and there is relatively strong linkage disequilibrium between these two genes, in this study the association of MICA mismatches with worse graft survival was HLA-independent and HLA-B independent. The clinical use of non-HLA antibody analytics is still somewhat controversial and routine monitoring is not recommended. Perhaps the best characterized phenotype is with AT1R-antibodies, and in case HLA-DSA-negative ABMR is detected after kidney transplantation, AT1R-antibodies could be measured and therapy with angiotensin receptor blockade considered in case of detection of antibodies [12]. It should be noted, however, that microvascular inflammation in the kidney transplant can be based on other mechanisms as well, and not only antibody-mediated. In addition to their possible clinical applications, non-HLA antibody targets could potentially be candidates for future genome-level matching studies.

## Candidate Gene Analyses

Early attempts to identify the association between non-HLA genetic variance and transplant outcomes come from studies that focus on candidate gene analyses, such as known polymorphisms in genes that influence inflammatory responses or drug metabolism [15, 16]. They have mostly focused on recipient genetic variation, as many cohorts may not have donor DNA available for analyses. Many studies are limited by small sample size, focusing on a single polymorphism or gene only, and lack external validation cohorts. Table 1 summarizes some studies of candidate gene – analyses. One of the best characterized single-gene associations with graft survival is genetic variations in apolipoprotein L1 (APOL1). Risk genotypes in *APOL1* in African Americans have been associated with the increased risk of end-stage kidney disease (ESKD) in native kidneys [30], and *APOL1* risk alleles in deceased donors have similarly been identified as a risk factors for graft loss [26, 27]. The association is thought to be mediated via kidney-related mechanisms of *APOL1* risk genotypes, but a recent study in African American recipients showed that also recipient risk alleles are associated with a higher risk of graft loss and identified immunomodulatory mechanisms behind this association [25]. Another interesting genetic aspect is the genetic findings seen in Natural Killer (NK) cell functionalities, which are involved especially in AMR responses. KLRC2 gene deletion variants, which determine the activating receptor NKG2C expression, were associated with microvascular inflammation and AMR-associated gene expression patterns, but the findings observed in a highly selected cohort of DSA positive patients did not translate into graft survival differences in a large multicenter cohort [28, 31]. In another study, FCGR2C Q^13^ in addition to FCGR3A V^176^ was a significant risk allele that could enhance NK cell-mediated antibody-dependent cellular cytotoxicity and contribute to allograft injury and poor survival [29]. Unfortunately, replication studies with large cohorts are missing for most of the reported associations. As positive candidate gene results have not been confirmed in the genome wide associations studies (below), there may also be publication bias toward positive findings. A systematic multi-cohort replication study of the reported associations is clearly warranted for critical evaluation of these results. If validated and proven predictive of higher risk of graft loss, identifying risk genotypes would possibly allow closer follow-up of risk patients, or help in improving organ allocation.

**TABLE 1:** Selected studies of candidate gene analyses and kidney transplant outcomes.

Gene/SNP, year	Clinical outcome	Number of patients analyzed	Main finding
Recipient TNFA, IL-10 [15], 1999	Rejection episodes, rejection severity, steroid responsiveness	100	TNF-alpha and IL-10 genotypes were associated with the risk of multiple rejection episodes or steroid-resistant rejection
Recipient CYP3A5 [16], 2003	Tacrolimus dose	80	CYP3A5 genotype predicted tacrolimus dose
Recipient TCF7L2 [17], 2009	New-onset diabetes after transplantation (NODAT)	1,076	Out of 11 polymorphisms, TCF7L2 was independently associated with NODAT
Recipient Exploratory analysis [18], 2010	Acute rejection	990	15 candidate SNPs identified, which were associated with acute rejection and 15 SNPs identified, which were associated with severity of tubulitis
Recipient IFN-gamma [19], 2010	Acute rejection, chronic allograft nephropathy	74	IFN-gamma genotype was associated with acute rejection and chronic allograft nephropathy
Recipient FOXP3 [20], 2013	Acute rejection, death-censored allograft loss	599	FOXP3 genotype was associated with allograft survival
Donor CAV1 [21], 2010	Death-censored graft failure	785 donors discovery, 697 donors validation	the CAV1 rs4730751 SNP was associated with allograft failure
Donor ABCB1, Donor CAV1 [22], 2015	Death-censored graft survival	682 donors, 1,233 recipients	ABCB1 was associated with shorter graft survival
Recipient LIMS1 [23], 2021	TCMR, ABMR, allograft survival	841	LIMS1 risk genotype associated with increased risk of TCMR
Recipient APOL1 [24], 2021	Long-term allograft outcomes	119	Among African-american recipients no association detected with APOL1 genotype and transplant outcomes
Recipient APOL1 [25], 2021	TCMR, death-censored allograft loss	507	APOL1 risk alleles were associated with death-censored graft- loss and TCMR
Donor APOL1 [26],2011	Graft survival	106 donors, 136 recipients	APOL1 risk alleles in African-American deceased donors were associated with higher risk of graft failure
Donor APOL1 [27], 2015	Graft survival	368 donors, 675 recipients	APOL1 risk alleles in African-American deceased donors were associated with higher risk of graft failure
Recipient KLRC2^wt/del^ variants [28], 2022	MVI, Graft survival	86 DSA+ recipients, and 1860 randomly selected recipients	KLRC2^wt/wt^ was associated with MVI in the smaller cohort, but no association with graft survival
Recipient FCGR3A^V/F158^, KLRC2^wt/del^, KLRK1^HNK/LN^ rs9916629-C/T	MVI, Graft survival	86 DSA+ recipients	Only KLRC2^wt/wt^ was associated with MVI, but not graft survival
Recipient FCGR2C Q^13^/STP^13^ and FCGR3A V^176^/F^176^ [29], 2024	MVI, Graft Survival	242 recipients	Q^13^ and V^176^ were associated with worse graft survival, and Q^13^ with MVI

ABCB1, multidrug resistance protein 1; CAV1, Caveolin-1; ATR, serine/threonine kinase; APOL1, apolipoprotein L1; FOXP3, forkhead box P3; IFNG, interferon gamma; IL15RA; LIMS1, LIM and senescent cell antigen-like-containing domain protein 1; SNP, single nucleotide polymorphism; TNFA, tumor necrosis factor alpha or TNF-α; TCF7L2, transcription factor 7-like 2; MVI, microvascular inflammation.

## Genome-Wide Association Studies (GWASs)

In contrast to single or candidate gene analyses, genome-wide association studies (GWAS) present an alternative approach to identify genetic variations associated with the outcome of interest [32]. With GWAS, millions of SNPs scattered across the genome can be analyzed. GWAS studies in other diseases have been useful in identifying novel risk factors and new mechanistic pathways [33–36], but the success of GWAS studies in the field of transplantation has been quite limited, mostly due to lack of adequately powered cohorts and lack of external validation and replication of the findings. As millions of SNPs are studied and as the study cohorts have heterogeneity in diagnoses and transplant procedures and treatments, thousands of recipient-donor pairs are needed for a statistically sufficient power. Usually associations with p-values clearly below 10exp-8 are regarded as indicative. Table 2 summarizes some GWAS studies within the field of kidney transplantation.

**TABLE 2:** Selected genome-wide association studies in the field of kidney transplantations.

Associated gene/SNP	Clinical association	Sample size and cohort type
TRA, ZNF516 [37]	Long-term graft function and survival	326 recipients, discovery
Validation of TRA and ZNF516 (see above), no association confirmed [38]	Death-censored graft loss, all-cause mortality	1,638 recipients, discovery
ATP5F1P6 [39]	New-onset diabetes after transplantation	256 recipients, discovery and 441 validation
LINC00882, CACNA1D, CSMD1 [40]	Cutaneous squamous cell carcinoma after transplantation	388 recipients, discovery
CYP3A5*6, CYP3A5*7 [41]	Tacrolimus pharmacokinetics	197 recipients, discovery and 160 validation
PTPRO, CCDC67 [42]	Acute rejection	778 recipients, discovery and 844 validation
No association detected outside HLA loci [43]	Long- and short-term allograft survival	2094 recipients and donors, discovery and 5,866 validation
41 donor SNPs found that contributed independently or interacted with APOL1 [44]	Death-censored graft survival	532 AA donors (978 recipients) discovery, 250 AA donors (465 recipients) validation

ATP5F1P6, ATP, synthase, H+ transporting, Mitochondrial Fo Complex, Subunit B1 Pseudogene 6; CACNA1D, Calcium Voltage-Gated Channel Subunit Alpha1 D; CCDC67, Coiled-Coil Domain-Containing Protein 67; CSMD1, CUB And Sushi Multiple Domains 1; CYP3A5, cytochrome P450; LIMS1, LIM and senescent cell antigen-like-containing domain protein 1; LINC00882, Long Intergenic Non-Protein Coding RNA 882, PTPRO, Protein Tyrosine Phosphatase Receptor Type O; TRA, T-cell receptor alpha; ZNF516, Zinc Finger Protein 516; ZSCAN25, Zinc Finger And SCAN, Domain Containing 25.

The first recipient-only GWAS for kidney allograft survival included 326 European transplants [37], and the study found two variants of interest showing genome-wide significance. The identified SNPs were independently associated with long-term graft function and survival. A larger study tried to confirm these findings in a multicenter cohort but failed to show any association of the identified SNPs with graft survival [38].

Another recipient GWAS analyzed 275 European cases of T-cell-mediated rejection and 503 controls, identifying five candidate loci. In a validation cohort of 313 cases and 521 controls, two loci remained significantly independently associated with acute rejection [42]. One locus encompassed *PTPRO* gene, coding for a receptor-type tyrosine kinase essential for B cell receptor signaling, the other ciliary gene *CCDC67*, essential in the functions of immune synapse and primary cilium. These functionally interesting findings could, however, not be validated in a large external cohort [45]. The largest genome-wide association study published to date to our knowledge involved 2,094 kidney transplant recipients and their donors, and a validation cohort of 5,866 pairs. This study could not find any strong donor or recipient genetic associations outside HLA region contributing to long- or short-term allograft survival [43].

These discrepancies in findings highlight the importance of adequate validation of exploratory findings in different cohorts, preferably with a different genetic background. Both kidney transplant rejection and graft failure are highly multifactorial; both recipient and donor characteristics, many perioperative events in both donor and recipient, and many posttransplant events modify the alloimmune risk, graft function, and survival prognosis, and it would require very extensive multicontinental cohorts to show the association of any weak genetic signals. Based on the published evidence so far, it seems unlikely that single genetic polymorphism loci could be identified that would largely explain variation in graft outcomes.

## Polygenic Risk Scores

As the effect of any individual single gene variation on transplantation outcome can be assumed to be very small, polygenic risk score (PRS) type of summary statistics may also prove to be informative in risk assessment. Polygenic risk scores (PRS) utilize and combine the existing GWAS findings to determine disease risk based on genetic variance [46]. With PRS, genome-wide genotype data are computed into a single variable of individual-level risk score. PRS approach has already been applied to variety of traits for common diseases. For example, a study by Khera et al [47] developed and validated PRS for five common diseases, showing that highest PRSs for complex multifactorial diseases identify risk levels close to those typically seen in single-gene Mendelian diseases. Their approach identified 8.0%, 6.1%, 3.5%, 3.2%, and 1.5% of the population at greater than three-fold increased risk for coronary artery disease, atrial fibrillation, type 2 diabetes, inflammatory bowel disease, and breast cancer, respectively.

PRS approach has also been studied for the risk of chronic kidney disease (CKD). Khan et el. combined *APOL1* risk genotypes with GWAS data of kidney function, and designed, optimized, and validated a PRS for CKD. The PRS was then tested in 15 independent cohorts, including 3 cohorts of European ancestry (n = 97,050), 6 cohorts of African ancestry (n = 14,544), 4 cohorts of Asian ancestry (n = 8,625) and 2 admixed Latin American cohorts (n = 3,625). The top 2% of the PRS was associated with nearly threefold increased risk of CKD across ancestries [48]. Attempts have also been made to predict native kidney function employing PRS approach. However, the ability of these scores to explain phenotypic variance in kidney function in native kidneys has been limited, e.g., below <4% in a study by Gorski et al [49].

Application of polygenic risk scores could be of potential value when evaluating the association of genetic variance with kidney transplant function or survival. To date, relatively few PRS studies in the field of kidney transplantation have been performed to our knowledge (Table 3).

**TABLE 3:** Selected polygenic risk score (PRS) studies in the field of kidney transplantations.

PRS trait	Clinical outcome	Sample size and cohort type	Main finding
Non-transplant NMSC, SCC and BCC [50]	Time to post-transplant NMSC	899 kidney recipients	PRS for non-transplant NMSC was predictive of case/control status and time to post-transplant NMSC
Posttransplant eGFR [51]	1‐year and 5‐years after transplantation, and change between 1 and 5 years eGFR	10,844 kidney recipients and donors	32% of the variability in eGFR at 1-year was explained by the model, with only 0.3% contributed by the PRS
Non-transplant BCC and SCC [52]	Post-transplant BCC and SCC	1,272 kidney recipients	PRS improves prediction over traditional skin cancer risk factors by 3% for BCC, but not for SCC
Non-transplant type 2 diabetes [53]	New-onset diabetes after transplantation	2,062 kidney recipients and 533 donors; 1,581 liver recipients and 1,555 donors	Recipient T2D PRS was associated with pre-transplant T2D and the development of PTDM. Combined liver donor and recipient T2D PRS improved PTDM prediction > 5% compared to a model with only clinical characteristic
Hypertension, stroke, and intracranial aneurysms (IA) [54]	Deceased donor age of death, graft function after transplantation	6,666 donor-recipient pairs	Donor PRS for hypertension was associated with reduced long term graft survival, and donor PRSs for hypertension and intracranial aneurysm were associated with reduced recipient eGFR at 1 year

BCC, basal cell carcinoma; eGFR, estimated glomerular filtration rate; NMSC, non-melanoma skin cancer; SCC, squamous cell carcinoma; T2D, type 2 diabetes; PTDM, posttransplant diabetes mellitus.

The largest PRS study in the field of kidney transplantations analyzed the association of PRS, calculated using genetic variants associated with non-transplant eGFR, with posttransplant eGFR in a cohort of 10,844 donor-recipient pairs [51]. The polygenic risk score was applied on both donors and recipients to predict kidney transplant function at 1 and 5 ears, in addition to change in eGFR post-transplantation. In this study, PRS calculated using the recipient’s genotype alone, as well as combined donor and recipient genotypes were significantly associated with eGFR at 1-year posttransplant. When the donor-recipient PRS was combined with clinical predictors of graft function and principal components, 32% of the variability in 1-year estimated GFR could be explained by the model. However, only 0.3% of the variation was contributed by the PRS. None of the PRS were significant predictors of graft function at 5 years.

In another study, Stapleton *et al.* examined the risk of non-melanoma skin cancer (NMSC) among 889 European ancestry kidney transplant recipients [50]. Genetic variants from previously published squamous cell carcinoma (SCC) and basal cell carcinoma (BCC) non-transplant GWAS was used for PRS, which was shown to be predictive of NMSC status and time to NMSC post-transplant.

Seviiri *et al.* generated PRSs from the general population, and studied whether these PRSs could predict and stratify the risks of BCC and SCC in a cohort of 1,272 solid-organ transplant recipients [52]. In this study, PRS was independently associated with both BCC and SCC. However, when combined with traditional skin cancer risk factors, no additive predictive value of PRS was seen in SCC, and in BCC the prediction was improved only with 3%.

A study by Shaked and colleagues looked for the association of post-transplant diabetes mellitus (PTDM) with type 2 diabetes (T2D) PRS in 1,581 liver recipients and their 1,555 donors, and 2,062 kidney recipients and 533 donors [53]. They examined whether recipient genomics contribute to PTDM development and were able to demonstrate that recipient T2D PRS is a predictor of PTDM, independently of known clinical predictors of PTDM.

A recent study by Collins *et al.* employed a multicenter dataset of 6,666 deceased and living kidney donors from 7 different European ancestry transplant cohorts, and investigated the association of polygenic risk scores for cerebrovascular disease risk factors [hypertension, stroke, and intracranial aneurysm (IA)] on deceased donor age of death, and kidney graft survival, and graft function [54]. Deceased kidney donors had an elevated genetic burden for hypertension and IA compared to living donors and healthy controls, and this burden was associated with donor age of death among donors who died of stroke. In addition, increased donor polygenic risk for hypertension and IA was associated with reduced graft function at 1 year [54].

As shown above, polygenic risk score approaches can be useful in defining the burden of genetic risk also after transplantation, but as expected by the various possible clinical scenarios and posttransplant events, the contribution of genetic risk in predicting posttransplant events remains limited and is not yet ready for clinical use. PRS approaches for predicting higher risk for cancer or acute rejection could be applied in the clinical setting for example for mandating closer follow-up (e.g., for the risk of skin malignancies), or tailoring individualized immunosuppression. One of the main problems with the PRS approaches is, however, that although they can possibly identify effectively the small cohort of patients at highest risk, they usually fail to identify the majority of the patients who experience the particular event of interest.

## Genome-Wide Mismatch Studies

Matching of HLA alleles is the golden standard in kidney transplantation. However, any genetic differences, or mismatches, in proteins expressed in the transplanted kidney can be recognized as immunologically foreign, leading to immune activation and increased risk of rejection. With the emergence of powerful genome analysis tools, these differences can now be identified. Mismatches in these so-called minor histocompatibility antigens have also been hypothesized to increase the risk of graft rejection and failure. In fact, cumulatively the minor histocompatibility antigens constitute a much larger pool of genetic differences between the donor and the recipient. Martin *et al.* were one of the first to use genome-wide single nucleotide polymorphism (SNP) arrays to predict amino acid differences between hematopoietic stem cell transplantation (HSCT) donors and recipients based on 19,104 coding SNPs. In HLA-matched sibling transplants, mismatches in coding SNPs were associated with an increased risk of graft-versus-host disease [55].

The role genome-wide non-HLA mismatches between kidney donor and recipient has also been studied after kidney transplantation. The study by Mesnard *et al.* was one of the first to analyze the importance of non-HLA donor-recipient mismatch in a cohort of 53 kidney transplantation donor-recipient pairs. They performed exome-sequencing for kidney transplant recipients and their living donors and estimated all the cell surface protein mismatches for each donor-recipient pair by calculating the number of amino acid mismatches in transmembrane proteins. This allogenomics mismatch score was predictive of long-term graft function, independent of HLA-A, -B, and -DR matching [56]. Pineda *et al.* tested the role of non-HLA donor-recipient mismatches in rejection in a cohort of 28 pairs, using exome-sequencing and gene expression data. They identified 123 non-HLA variants associated with mainly antibody-mediated rejection processes [57].

In a study by Reindl-Schwaighofer *et al.*, genome-wide mismatches in nonsynonymous (amino acid changing) SNPs (nsSNPs) were evaluated among 477 genotyped kidney transplant recipients and their deceased donors. The degree of nsSNP mismatch in transmembrane and secretory proteins, adjusted for HLA eplet mismatch, was independently associated with graft loss. Furthermore, customized peptide arrays were used to verify a donor-specific alloimmune responses to genetically predicted mismatched epitopes in a subset of 25 patients [58]. In a cohort of 385 donor-recipient pairs of multiethnic origins, Zhang *et al.* analyzed genetic differences between the donor and the recipient using genome-wide SNP array data, excluding the HLA region. They estimated the ancestry in each donor-recipient pair and proportion of genome-shared identity-by-descent (pIBD) between donor-recipient pairs. In donor-recipient pairs of similar ancestry, pIBD was significantly associated with graft survival, independent of HLA mismatches. In addition, pIBD was significantly associated with early vascular intimal fibrosis, which was an independent predictor of graft survival [59].

In addition to studying mismatches at SNP level, one interesting approach is to study gene deletions and their associations to kidney transplantation outcomes. It is shown that some gene deletions are common among population [60]. Individuals who inherit both deleted alleles from their parents lack the functional gene and the protein product. When such an individual receives a graft from a donor who carries at least one functional copy of that gene, recipient’s immune system may recognize the protein as foreign*.* A study by Steers *et al.* tested this hypothesis in a discovery cohort of 705 kidney transplant recipients, and validated findings in a genomic collision model of 2004 donor-recipient pairs from three independent cohorts. Genomic collision was defined as a specific donor-recipient genotype combination, in which a recipient who was homozygous for a gene-intersecting deletion received a transplant from a nonhomozygous donor. They found that a homozygous variant rs893403, a marker for the deletion of LIMS1 gene, was associated with rejection independently of the HLA mismatch and other clinical factors. In addition, a specific antibody response against LIMS1 was identified [61].

In a recent study, Sun *et al.* performed a genome-wide SNP array for two prospective kidney transplant donor-recipient cohorts (385 and 146 pairs) with the goal of identifying mismatches within non-HLA loci that associate with long-term death-censored graft loss (DCGL). After first confirming that donor-recipient differences resulting from SNP mismatches associate with DCGL, they searched for the mismatches across all annotated gene loci, in order to identify individual gene-level mismatches that significantly associated with increased risk of graft loss. The screening confirmed *LIMS1* as a top-ranked gene locus associated with DCGL, independent of genome-wide mismatches. Interestingly, they demonstrated that rather than leading to alloimmune response against the missing LIMS1 protein, the deletion resulted in changes of expressions of certain other genes with functional effects related to the outcome. Hence, it is not clear whether the LIMS1 gene deletion leads to alloimmune response or other downstream functional effects [62].

In our own study, we analyzed the association of the sums of whole-genome missense variant mismatches and missense mismatches in transmembrane, secretory, and kidney-related proteins, with acute rejection in a single center cohort of 1,025 kidney transplant recipients and their deceased donors. We found that sums of kidney-related proteins were associated with the risk of acute rejection in unadjusted analyses. In deletion analysis, the previously detected association with *LIMS1* deletion and acute rejection could not be confirmed in our cohort. However, a mismatch in rs7542235 genotype GG tagging a homozygous deletion at the complement factor H-related (*CFHR*) proteins locus was independently associated with acute rejection [63]. We have then further characterized 15 patients with *CFHR* deletion of various sizes, and have found that the different deletion types share the complete deletion of the *CFHR1* gene pointing to its primary role. Plasma proteomics studies showed that deletion-tagging allele is associated with altered expression of CFH/CFHR proteins, and some other proteins as well [64]. Table 4 summarizes donor-recipient mismatch studies in the field of kidney transplantation. These studies very elegantly show that whole genome mismatch concept has proven useful in identifying novel targets of alloimmunity outside the HLA region, and may provide mechanistic targets for future studies and drug development. In addition, these findings could be applied in the clinical setting for individualizing immunosuppression or follow-up for the highest risk groups.

**TABLE 4:** Donor-recipient genetic mismatch studies in the field of kidney transplantations.

Mismatches studied (found gene)	Clinical outcome	Sample size and cohort type	Main findings
Amino-acid mismatches in transmembrane proteins [56]	Long-term allograft outcome measured by eGFR	53 donor-recipient pairs	Allogenic mismatch score was independently associated with posttransplant eGFR
All genome-wide mismatches [57]	ABMR, TCMR	28 donor-recipient pairs	non-HLA mismatch variants were associated with AMR
nsSNPs mismatches in transmembrane and secretory proteins [58]	Graft loss	477 donor-recipient pairs	The degree of nsSNP mismatch was associated with graft loss, independently of HLA incompatibility
Mismatches in genome-shared identity-by-descent SNPs [59]	Death-censored allograft loss	385 donor-recipient pairs	Genome-shared identity-by-descent was associated with graft survival, independent of HLA mismatches, and with early vascular intimal fibrosis
50 deletion-tagging SNPs (LIMS1) [61]	Kidney allograft rejection	705 kidney transplant recipients and 2,004 donor-recipient pairs	Genomic collision at LIMS1 locus was associated with rejection and with production of anti-LIMS1 IgG2 and IgG3
Non-HLA mismatches at variant-, gene-, and genome-wide scales (LIMS1) [62]	Death-censored graft loss	385 and 146 donor-recipient pairs	Mismatch at the LIMS1 locus was associated with graft loss. The deletion resulted in changes of expressions of other genes with functional effects
Mismatches in kidney-related proteins, CFHR- deletion [63]	Acute rejection	1,025 recipient-donor pairs	Sums of kidney-related proteins were associated with acute rejection in unadjusted analyses. A mismatch in CFHR deletion was associated with acute rejection

ABMR, antibody-mediated rejection; eGFR, estimated glomerular filtration rate; nsSNP, nonsynonymous single nucleotide polymorphism; TCMR, t-cell mediated rejection.

## Conclusions and Future Directions

A common problem with genetic studies in clinical medicine is that the complex interplay of genetic and environmental factors in defining clinical outcomes requires extensive datasets to show any genetic signal to have meaningful association with outcomes. In addition, the genetic factors predisposing to disease states may be different among different populations. Most likely the genetic factors predisposing to disease are different to those regulating the disease progression. Transplantations have some additional challenges. The indications for transplantation can be fundamentally different disease entities from genetic diseases to autoimmune or metabolic diseases. Furthermore, even if PRS models or GWAs studies could identify individuals at higher risk of graft loss or acute rejection, most of these models apply only to the highest risk individuals (top 2%–5%), and fail in sensitivity to identify the large majority of the patients at risk for these events.

Importantly, the transplant outcome always depends on two individuals. The genetic properties of donor and recipient, and their combination or mismatch play a role in predicting outcomes after transplantation, adding further complexity. Therefore, international collaboration and preferably very large multicenter international consortia, such as the iGeneTRAiN^1^, are required to validate the findings, and are very important to have any large impact within our field. Although not directly related to genetics of the alloimmune response, the first clinical applications of genetic testing in transplantation come from the field of pharmacogenomics, where genetic variants were identified in thiopurine methyltransferase (TPMT), involved in azathiprione metabolism, predisposing patients to a risk of drug-induced myelosuppression [65, 66]. In addition, genetic variants in the *CYP*-metabolism system can be analyzed and this information can be used to define correct dosing of tacrolimus [67]. Future discoveries and development in genetic testing of alloimmunity could for example, allow identification of high-risk patients for closer clinical monitoring or individualized immunosuppression, or help optimizing organ allocation.

## Footnotes

1
http://igenetrain.org/
